# Supplementary material for: Decorin Protects Cardiac Myocytes against Simulated Ischemia/Reperfusion Injury
Source: Molecules. 2020 Jul 28;25(15):3426. doi: 10.3390/molecules25153426 (PMC7436189; doi:10.3390/molecules25153426)
Supplement: Supplementary file 1 [file molecules-25-03426-s001.pdf]

2020.01.22-23, GEL 1

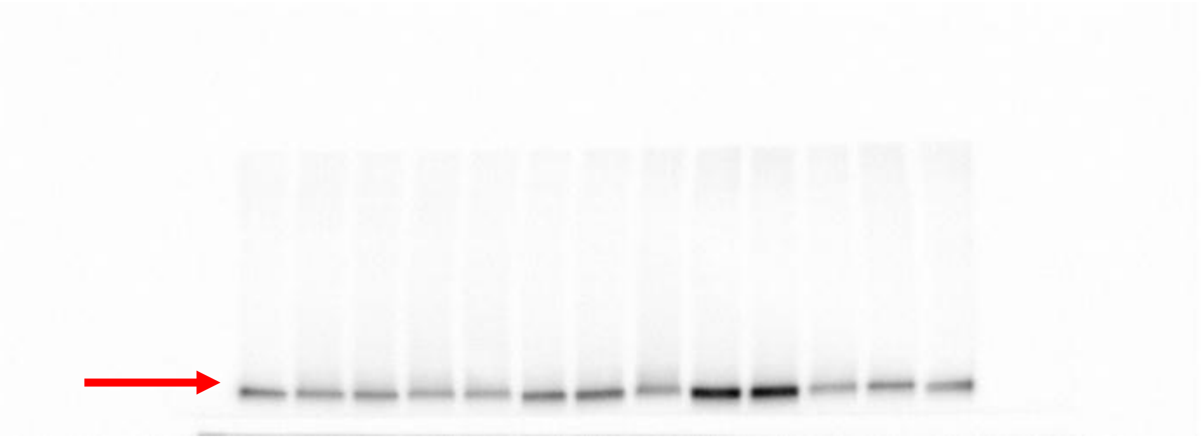

Akt:N_veh/2, N_1nm/1, N_3nM/1, N_10nM/1, N_30nM/1, H_veh/1, H_1nM/1, H_3nM/1, H_10nM/1, H_30nM/1, H_100nM/1, N_veh/1, H_veh/3

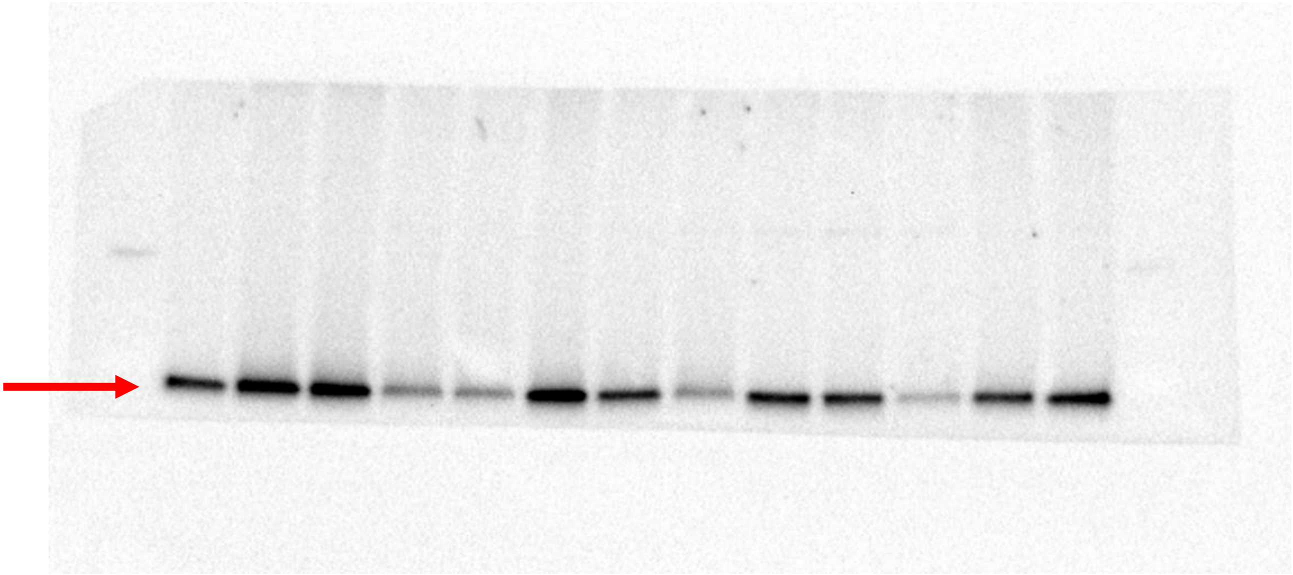

P-Akt:N_veh/2, N_1nm/1, N_3nM/1, N_10nM/1, N_30nM/1, H_veh/1, H_1nM/1, H_3nM/1, H_10nM/1, H_30nM/1, H_100nM/1, N_veh/1, H_veh/3

2020.01.22-23, GEL 1

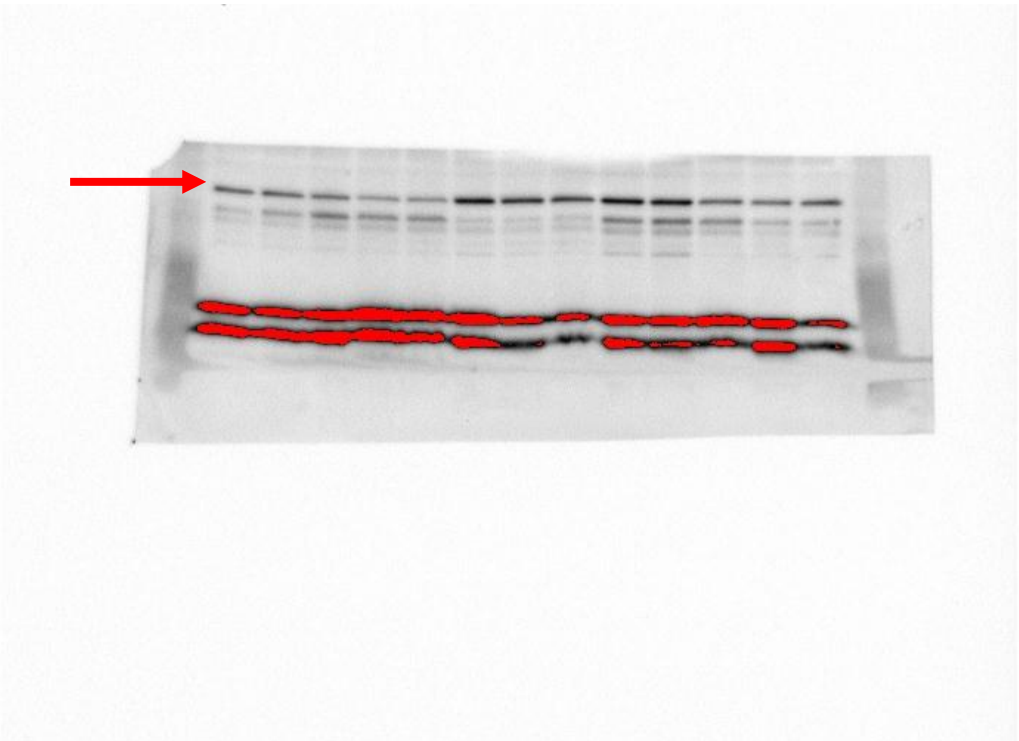

GAPDH:N_veh/2, N_1nm/1, N_3nM/1, N_10nM/1, N_30nM/1, H_veh/1, H_1nM/1, H_3nM/1, H_10nM/1, H_30nM/1, H_100nM/1, N_veh/1, H_veh/3

2020.01.22-23, GEL 2

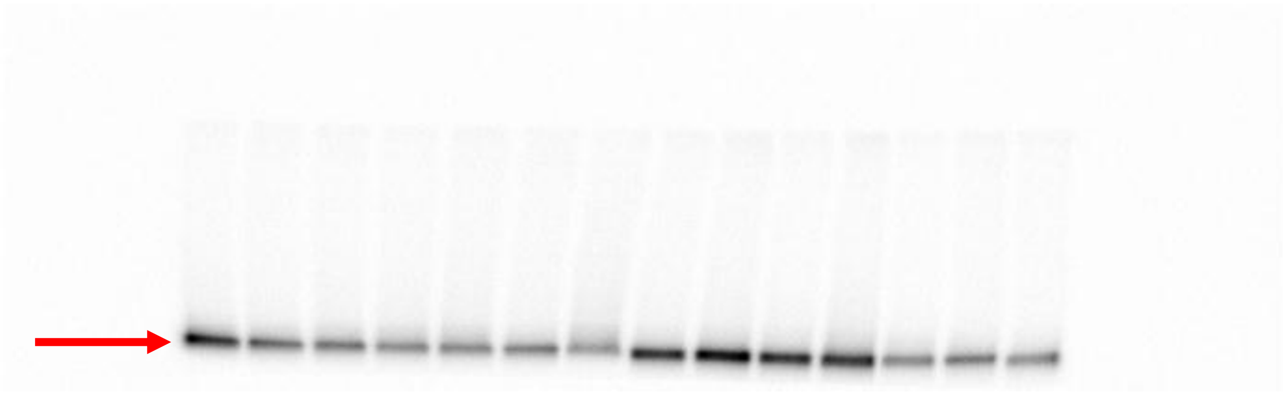

Akt:N_veh/3, N_1nm/2, N_3nM/2, N_10nM/2, N_30nM/2, N_100nM/2, H_veh/2, H_1nM/2, H_3nM/2, H_10nM/2, H_30nM/2, H_100nM/2, N_veh/1, H_veh/3

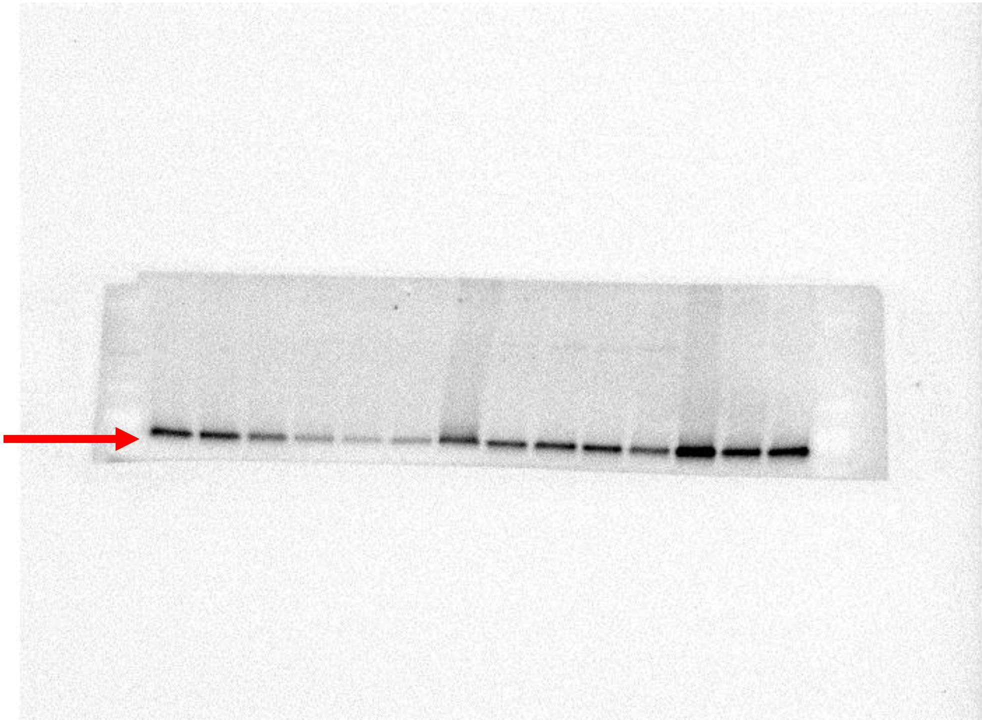

P-Akt:N_veh/3, N_1nm/2, N_3nM/2, N_10nM/2, N_30nM/2, N_100nM/2, H_veh/2, H_1nM/2, H_3nM/2, H_10nM/2, H_30nM/2, H_100nM/2, N_veh/1, H_veh/3

2020.01.22-23, GEL 2

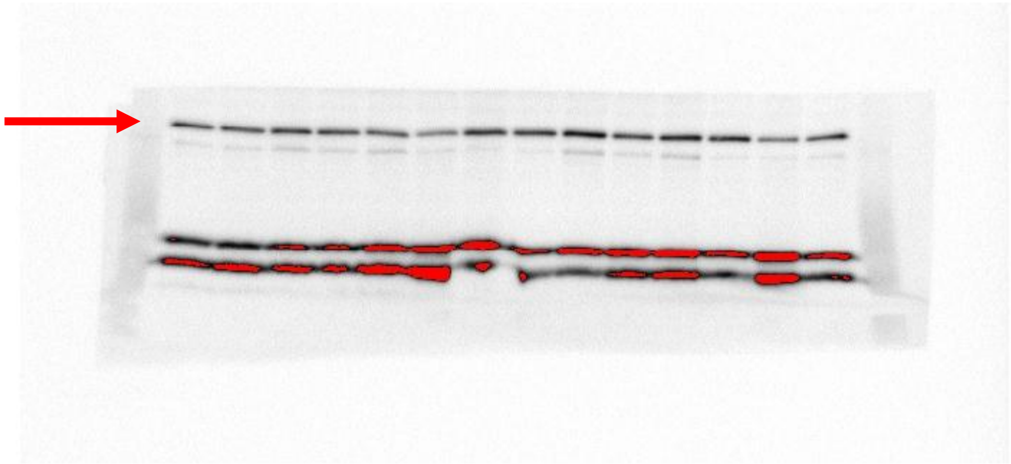

GAPDH:N_veh/3, N_1nm/2, N_3nM/2, N_10nM/2, N_30nM/2, N_100nM/2, H_veh/2, H_1nM/2, H_3nM/2, H_10nM/2, H_30nM/2, H_100nM/2, N_veh/1, H_veh/3

2020.01.22-23, GEL 3

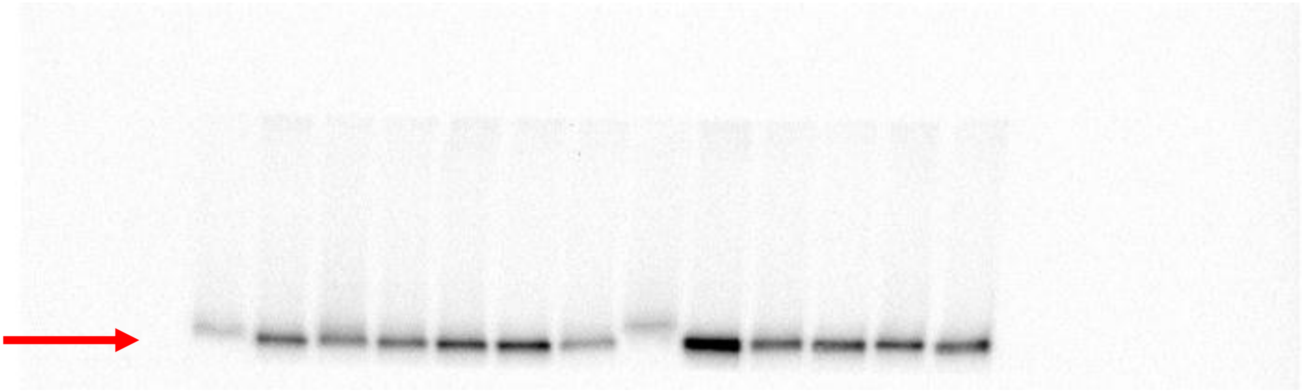

Akt:N_veh/4, N_1nm/3, N_3nM/3, N_10nM/3, N_30nM/3, N_100nM/3, H_veh/6, H_1nM/3, H_10nM/3, H_30nM/3, H_100nM/3, N_veh/1, H_veh/3

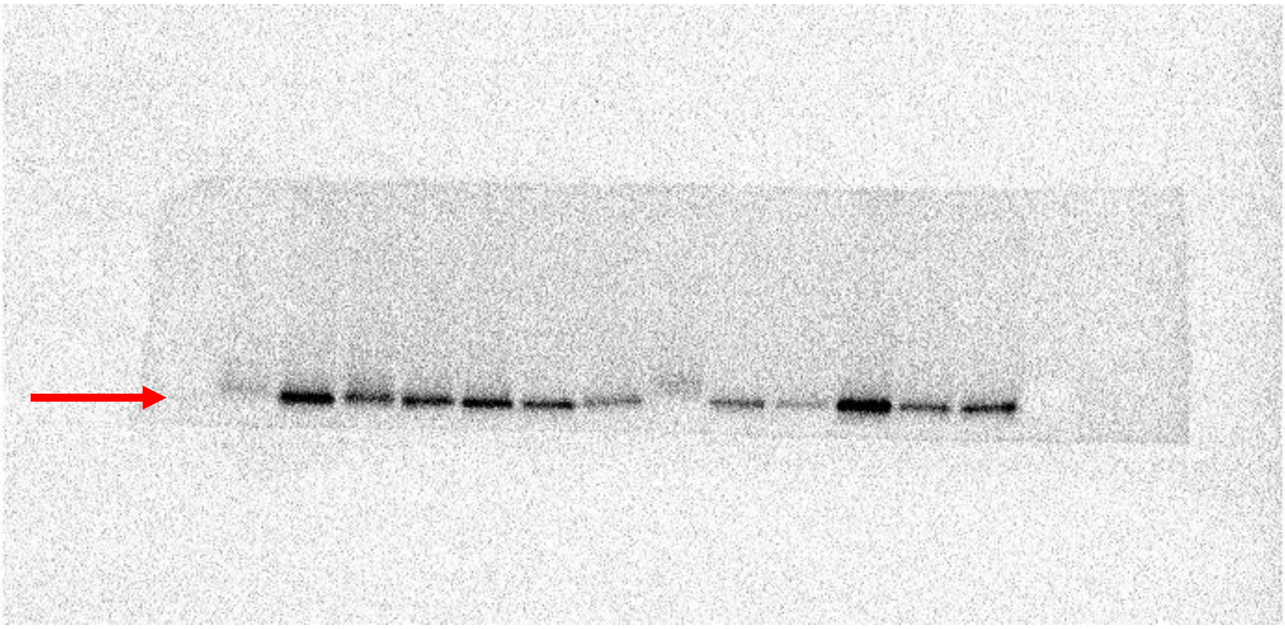

P-Akt:N_veh/4, N_1nm/3, N_3nM/3, N_10nM/3, N_30nM/3, N_100nM/3, H_veh/6, H_1nM/3, H_10nM/3, H_30nM/3, H_100nM/3, N_veh/1, H_veh/3

2020.01.22-23, GEL 3

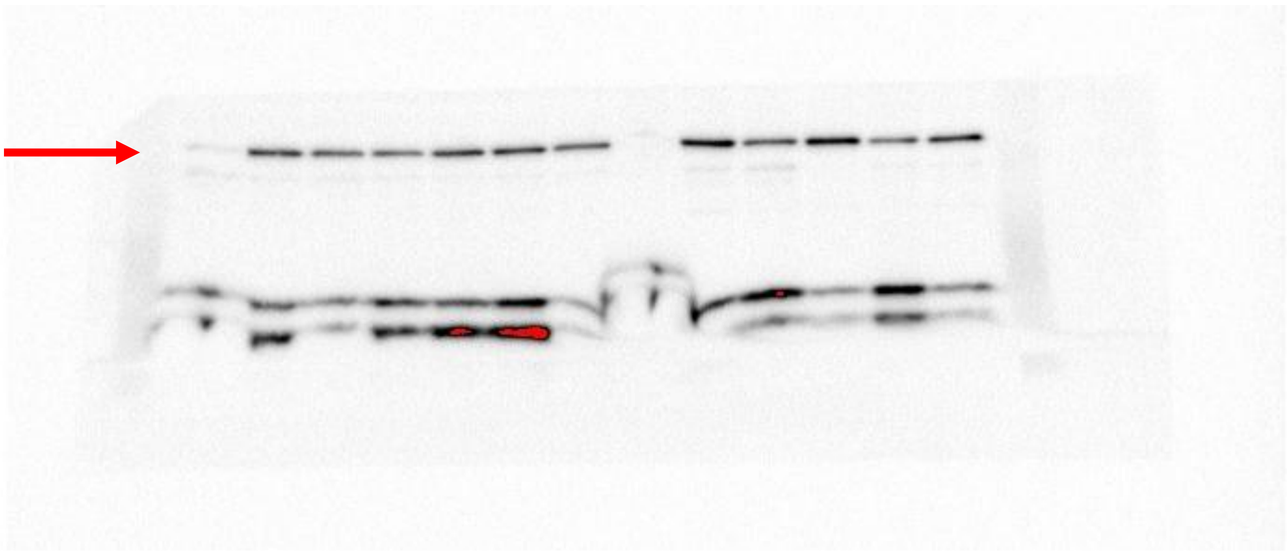

GAPDH:N_veh/4, N_1nm/3, N_3nM/3, N_10nM/3, N_30nM/3, N_100nM/3, H_veh/6, H_1nM/3, H_10nM/3, H_30nM/3, H_100nM/3, N_veh/1, H_veh/3

2020.01.22-23, GEL 4

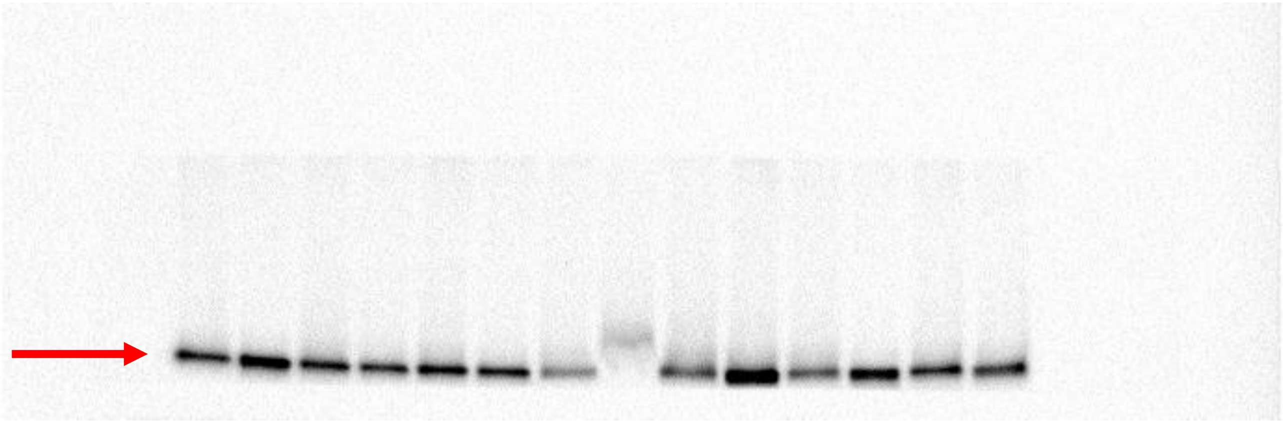

Akt:N_veh/5, N_1nM/4, N_3nM/4, N_10nM/4, N_30nM/4, N_100nM/4, H_veh/7, H_1nM/4, H_3nM/4, H_10nM/4, H_30nM/4, H_100nM/4, N_veh/1, H_veh/3

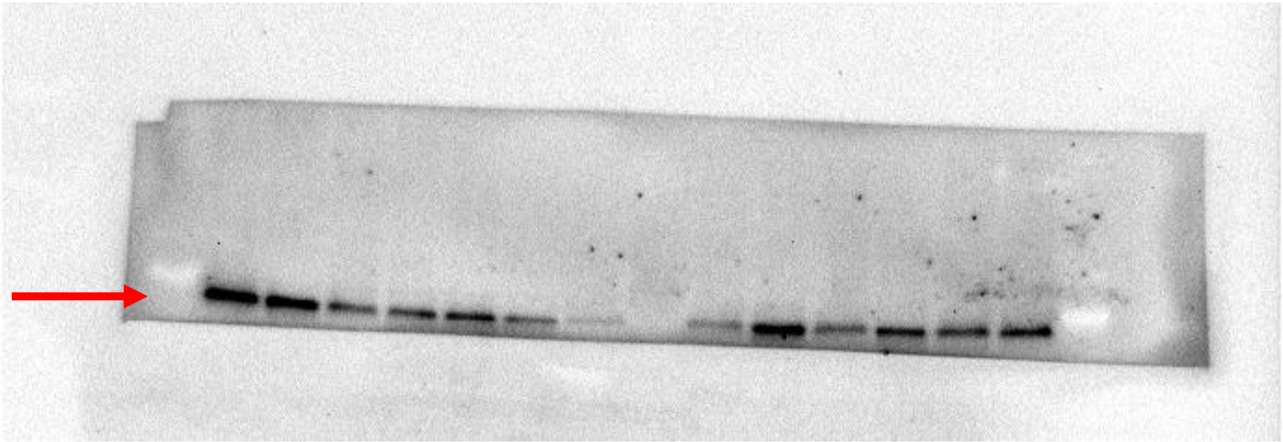

P-Akt:N_veh/5, N_1nM/4, N_3nM/4, N_10nM/4, N_30nM/4, N_100nM/4, H_veh/7, H_1nM/4, H_3nM/4, H_10nM/4, H_30nM/4, H_100nM/4, N_veh/1, H_veh/3

2020.01.22-23, GEL 4

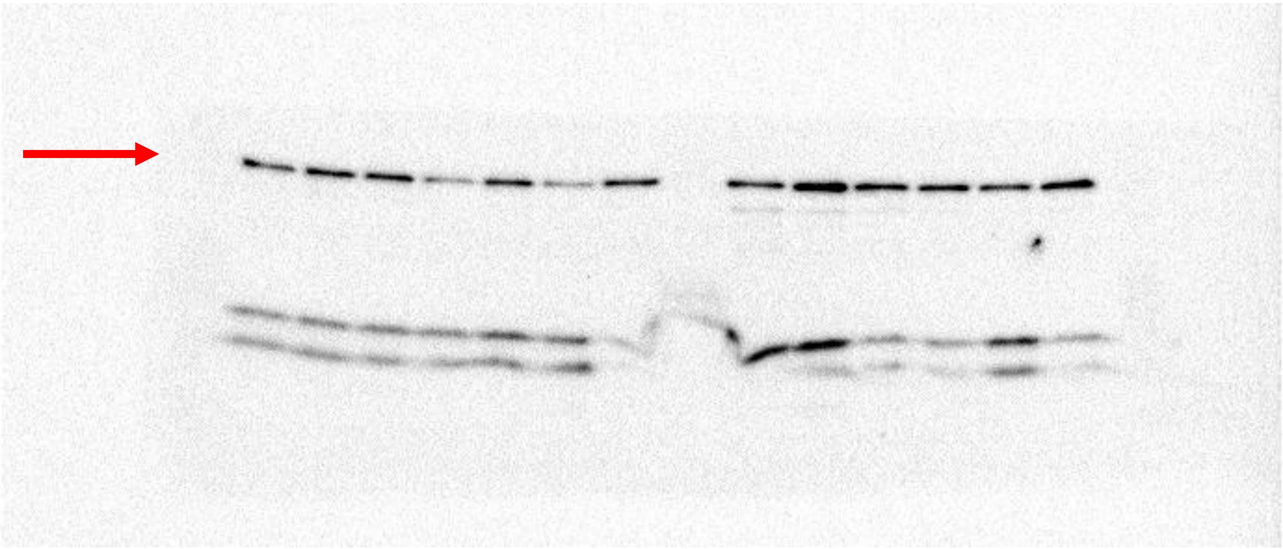

GAPDH:N_veh/5, N_1nM/4, N_3nM/4, N_10nM/4, N_30nM/4, N_100nM/4, H_veh/7, H_1nM/4, H_3nM/4, H_10nM/4, H_30nM/4, H_100nM/4, N_veh/1, H_veh/3

2020.02.13-14, GEL 5

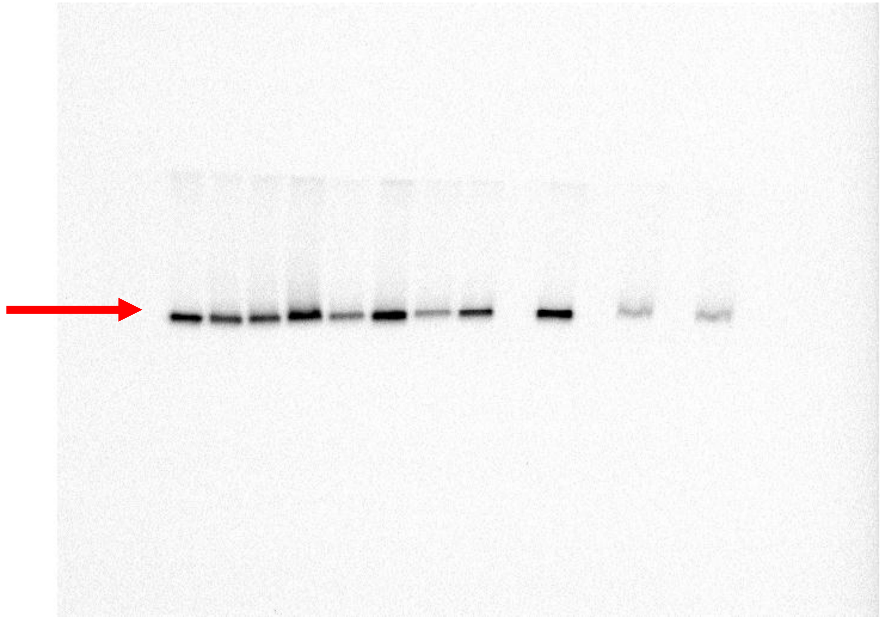

Akt: N_veh/1, H_veh/3, H_veh/2, N_veh/4, H_veh/6, H_10nM/3, H_veh/7, H_3nM/4, H_1nM/2, H_1nM/3, H_1nM/4

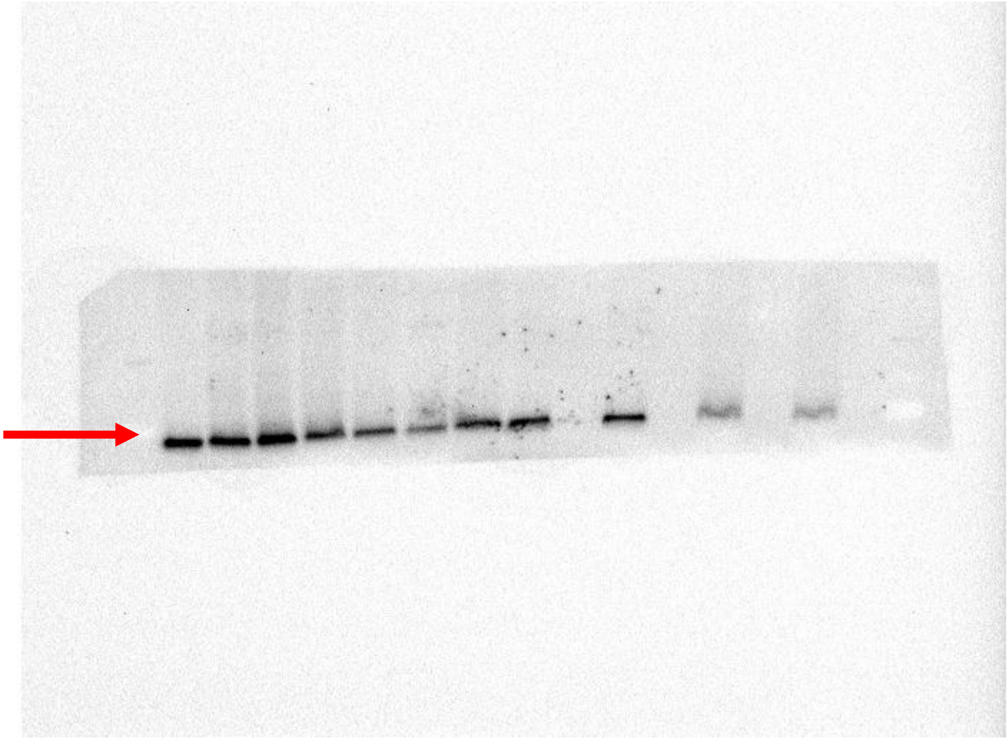

p-Akt: N_veh/1, H_veh/3, H_veh/2, N_veh/4, H_veh/6, H_10nM/3, H_veh/7, H_3nM/4, H_1nM/2, H_1nM/3, H_1nM/4

2020.02.13-14, GEL 5

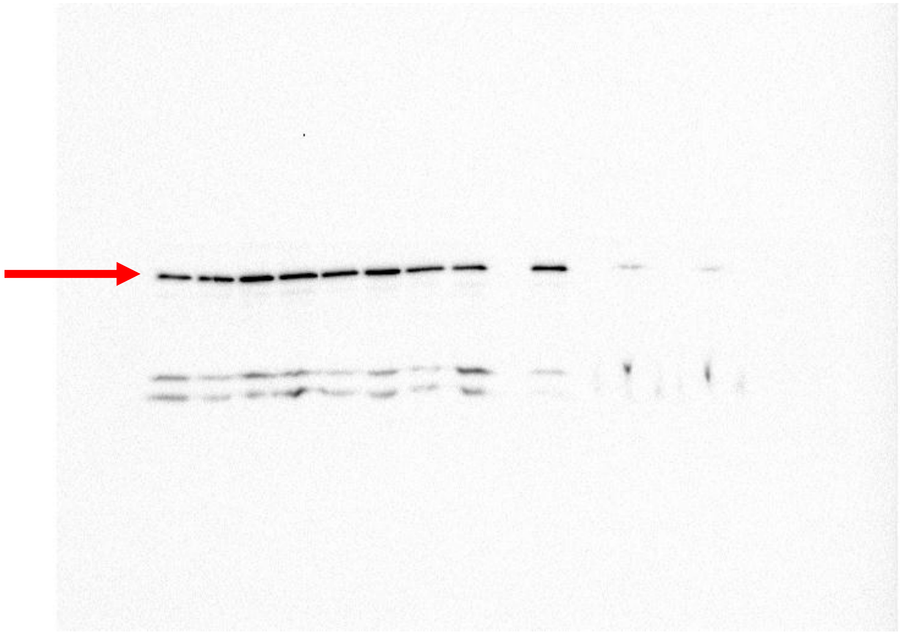

GAPDH: N_veh/1, H_veh/3, H_veh/2, N_veh/4, H_veh/6, H_10nM/3, H_veh/7, H_3nM/4, H_1nM/2, H_1nM/3, H_1nM/4
